# Identification of Communal Oviposition Pheromones from the Black Fly *Simulium vittatum*

**DOI:** 10.1371/journal.pone.0118904

**Published:** 2015-03-18

**Authors:** Tommy W. McGaha, Ryan M. Young, Nathan D. Burkett-Cadena, Joseph P. Iburg, Jeremy M. Beau, Sayed Hassan, Charles R. Katholi, Eddie W. Cupp, Bill J. Baker, Thomas R. Unnasch, Raymond Noblet

**Affiliations:** 1 Department of Entomology, University of Georgia, Athens, Georgia, United States of America; 2 Department of Chemistry and Center for Drug Discovery and Innovation, University of South Florida, Tampa, Florida, United States of America; 3 Global Health Infectious Disease Research Program, Department of Global Health, University of South Florida, Tampa, Florida, United States of America; 4 Department of Crop and Soil Sciences, University of Georgia, Athens, Georgia, United States of America; 5 Department of Biostatistics, University of Alabama at Birmingham, Birmingham, Alabama United States of America; 6 Department of Entomology and Plant Pathology, Auburn University, Auburn, Alabama, United States of America; Fundação Oswaldo Cruz, BRAZIL

## Abstract

The suite of pheromones that promote communal oviposition by *Simulium vittatum*, a North American black fly species, was identified and characterized using gas chromatography-mass spectrometry, electrophysiological, and behavioral bioassays. Behavioral assays demonstrated that communal oviposition was induced by egg-derived compounds that were active at short range and whose effect was enhanced through direct contact. Three compounds (*cis*-9-tetradecen-1-ol, 1-pentadecene, and 1-tridecene) were identified in a non-polar solvent extract of freshly deposited *S*. *vittatum* eggs that were capable of inducing the oviposition response. Electroantennography demonstrated that two of these three compounds (1-pentadecene and 1-tridecene) actively stimulated antennal neurons. Identification of the oviposition pheromones of this family may be helpful in developing control measures for nuisance black flies and for medically-important species such as *Simulium damnosum sensu lato*.

## Introduction

Conspecific aggregation among non-social insects occurs throughout the Class Insecta and is believed to confer a variety of fitness benefits [1]. Groups may form to exploit feeding or sheltering sites or for mating. Conversely, aggregation carries with it the risk of several deleterious outcomes such as competition for limited food resources as populations mature in number and size, and the rapid spread of pathogens that often occurs among densely grouped populations [1]. Stimuli that promote group joining are varied and range from the chemical and physical characteristics of the micro-environment in which aggregation occurs to the production of specific pheromones that induce joining behavior, i.e. chemicals secreted by an individual that evoke a specific behavioral response by a member of the same species.

Selection of suitable oviposition sites is also a crucial factor in population survival and the occurrence of conspecific, gravid females aggregating to deposit eggs occurs throughout the Insecta. Among hemi-metabolous insects, *Schistocerca gregaria* (the desert locust) exemplifies this behavioral trait, as gravid females are attracted to deposit egg masses communally by a combination of environmental and pheromone stimuli [2]. This type of pheromonal-directed reproductive behavior is seen also among more recently evolved, medically-important species within the Order Diptera. Some species of mosquitoes and sand flies oviposit communally and tsetse flies, which are oviparous, deposit larvae that aggregate shortly after deposition (reviewed by McCall and Cameron [3]). Chemical and behavioral evidence indicates that pheromones contribute to communal oviposition behavior, with erythro-6-acetoxy-5-hexadecanolide stimulating aggregation in *Culex quinquefasciatus* [4] and dodecanoic acid in *Lutzomyia longipalpis* [5].

Many black fly species in the genus *Simulium*, including members of the *Simulium damnosum* sensu lato species complex that transmit the human filarial parasite *Onchocerca volvulus*, (the causative agent of onchocerciasis, or river blindness) exhibit such a behavior as well, ovipositing in aggregations on a single substrate, producing clumped masses of fertile eggs [6–9]. The immature stages of black flies occur almost exclusively in lotic ecosystems and the identification and exploitation of patchy oviposition sites by gravid females is a key factor in ensuring population stability. Muirhead-Thompson [9], while observing ovipositing females in the *Simulium damnosum* complex, noted that multiple female flies were simultaneously depositing eggs on the same substrate and creating multi-layers of fresh ova. Further research with species in the *S*. *damnosum* complex led to the conclusion that communal oviposition was partially mediated by non-polar semiochemicals emitted from fresh eggs and originating in the ovaries during egg development [10–12]. However, unlike the situation for *Culex quinquefasciatus* and *Lutzomyia longipalpis*, the specific chemical compound(s) invoking this behavior or the mechanism through which these compounds act have not been determined.

In the studies described below, we have used electrochemical and behavioral assays to investigate semiochemical-mediated oviposition in black flies. The goal of the work was to test whether chemical compounds and crude extracts from egg masses would elicit communal oviposition behavior and to identify individual chemical constituents from egg extracts that induce oviposition using in choice assays. Experiments were conducted using a laboratory-colonized strain of *Simulium vittatum*, a North American black fly species that exhibits a communal oviposition behavior similar to the African black fly vectors associated with river blindness.

## Materials and Methods

### Source of Insects

Experiments were conducted with gravid *S*. *vittatum* (= cytospecies IS-7), reared in a colony at the University of Georgia as previously described [13]. Inseminated females were kept at 21–25°C and 70–90% relative humidity (r.h.) for 3–5 days to produce the gravid females used for behavioral and electroantennogram (EAG) experiments. Fresh 30 to 60 minute old eggs were obtained by allowing gravid *S*. *vittatum* to oviposit in an oviposition chamber for 30 minutes on filter paper circles (Whatman, No. 1, GE Healthcare, Little Chalfont, UK) for the behavioral experiments, or green linen (Jo-Ann’s Fabric Shops, Athens, GA, USA) for the chemical analyses. The number of eggs collected ranged from 500 to 2000.

### Preparation and Analysis of Egg Extracts

Collection of conspecific eggs for extraction was performed with a sterile razor by scraping off freshly deposited eggs from the linen substrate used in the oviposition chamber into a 2.0 mL amber glass vial (Wheaton Science Products, Millville, NJ, USA). Hexane and methanol were used as solvents for the extraction of *S*. *vittatum* eggs [5,10,11,14,15]. The vial, containing eggs and 1.5 mL of solvent, was agitated on a vortex mixer for 15 sec and placed at −60°C for 24 hours. The vial was allowed to thaw and the supernatant removed, filtered through glass wool and celite and placed in a clean vial. This crude extract was stored at −60°C until used for behavioral experiments or chemical analysis.

Chemical analysis of the crude hexane extract of freshly deposited eggs was initially performed with gas chromatography (GC) to identify peaks representing potential oviposition pheromones. Gas chromatography analyses were conducted on an Agilent 7980A GC interfaced to an Agilent 7000 series QqQ mass spectrometer (Agilent Technologies, Santa Clara, CA, USA) operating in electron ionization (EI) mode. Injections (2 μL) of the lipophilic extract solution from fresh eggs were vaporized on the preheated split-less inlet at 360°C and introduced onto an SLB-5ms column (30 m × 0.25 mm i.d., 0.25 μm film thickness, Supelco 28471-U) using a 20 m temperature gradient (initial oven temperature of 150°C, held for 4 minutes, heated to a final temperature of 230°C at a rate of 4°C/minute, then held at final temperature for a further 3 minutes). Helium was used as a carrier gas at a constant flow rate of 1 mL/m. Chemical species were identified by matching the mass spectra to known standards using the NIST/Wiley 2011 Database.

### Source of Identified Compounds Used in Experiments

**Fig 1:**
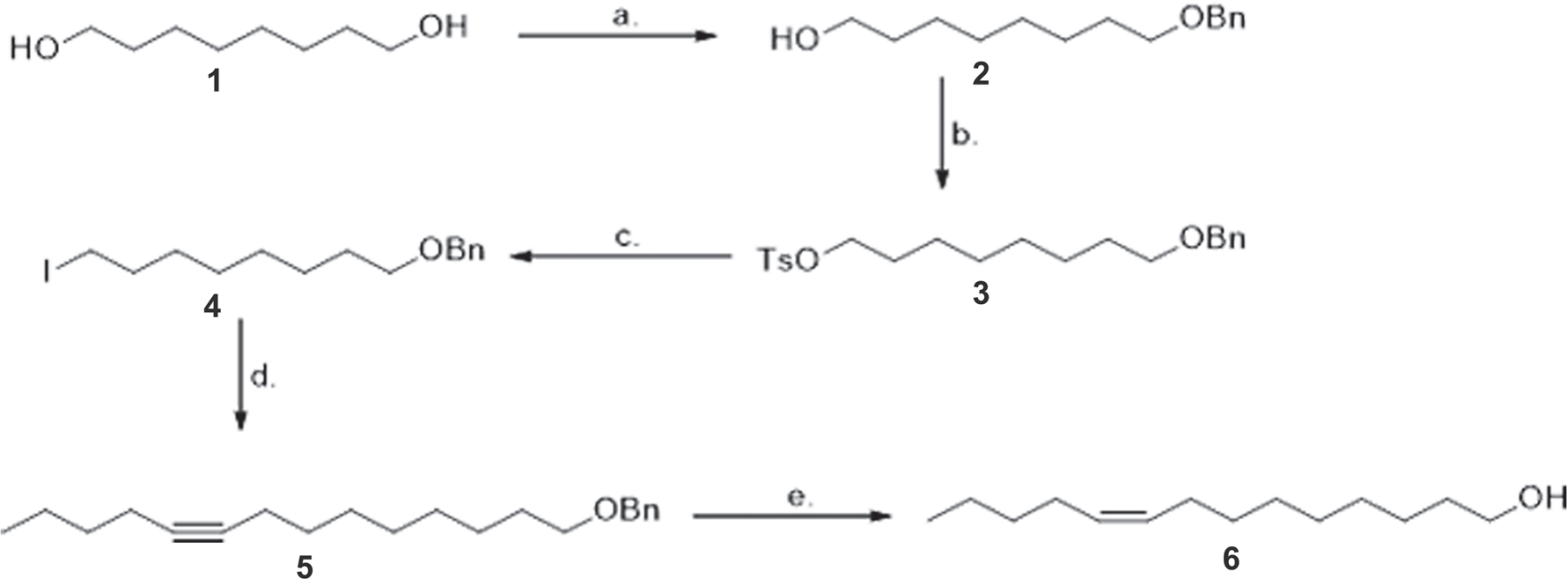
Synthesis of *cis*-9-tetradecen-1-ol. a. Ag_2_O, BnBr, DCM, RT, 18 h; b. TsCl, anhydrous pyridine, RT, 1 h; c. NaI, DMF, 50°C, 4 h; d. hexyne, *n*-BuLi, HMPA, THF, -78°C to RT, 96 h; e. Lindlar cat., quinoline, H_2_, 8 h. For more detail see S1 Text.

1-Pentadecene (97%), 1-hexadecene (94%), and 1-tridecene (97%) were obtained from Alfa Aesar (Ward Hill, MA, USA). *cis*-9-Tetradecen-1-ol was synthesized *de novo*. The synthesis procedure is presented schematically in Fig. 1 and the details of the synthesis procedure are presented in S1 Text.

### Behavioral Assays

All of the behavioral bioassays were conducted in a climate-controlled room at 23–26°C and 75–90% r.h. The room was completely dark with the exception of the light emitted from the behavioral bioassay light sources. Gravid females were used for all experiments.

**Fig 2:**
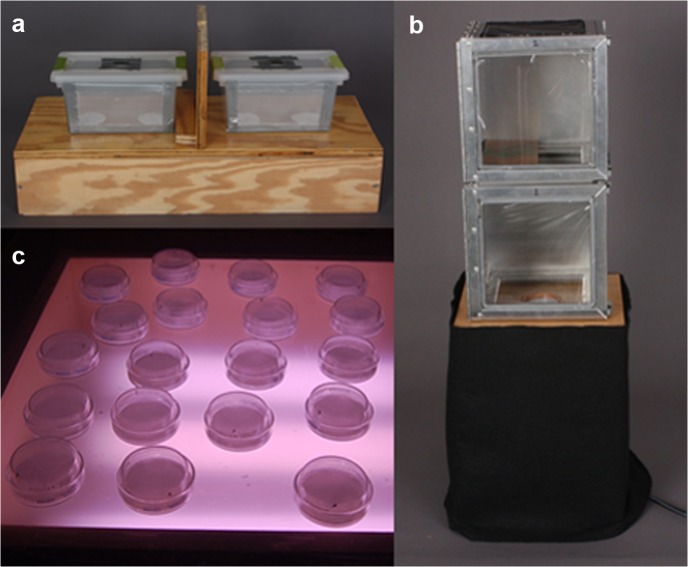
Behavioral bioassay apparatuses used in this study. Panel a: binary choice chamber; Panel b: orientation double chamber, with oviposition substrate uncovered; Panel c: Petri dish assay.

The binary choice chamber bioassay (Fig. 2, Panel a) was initially used to assess the behavior of gravid *S*. *vittatum* when given the choice of two oviposition substrates, one with no eggs and one with eggs. This bioassay was based on previous experiments that involved observing the oviposition preference of gravid *S*. *damnosum s*.*l*. and *S*. *ochraceum s*.*l*. [12,16]. The binary choice chamber was a modified 27.9 x 16.8 x 13.7 cm polystyrene box (Sterilite Corporation, Townsend, MA, USA). A 2.5 x 2.5 cm square opening was cut and lined with dental dam, providing an opening for introducing flies into the chamber. Treated filter paper was placed inside the chamber on top of two 4.5 cm holes, which were 13.0 cm apart (center to center). The plastic front of the chamber was removed and replaced with plastic mesh, which had 1.0 cm vertical slits cut in a perpendicular position to the center of the holes, to allow removal of the flies during the experiment. A wooden light box with a polyurethane coated surface served as the base for the binary choice chamber. The wooden box was 73.0 x 30.5 x 12.7 cm with two sets of paired holes; each pair of holes represented a place for one binary choice chamber. The inside of the wooden box was lined with aluminum foil to evenly distribute light through the holes of the binary choice chamber. The white light source inside the box was a 56 cm fluorescent light (T8 plant growth, Utilitech, distributed by Good Earth Lighting Inc., Wheeling, IL, USA) that rested on a sheet of aluminum foil. The light intensity emitted from the holes was determined to be 57.7 ± 7.5 foot-candles when measured with a photometer (Photometer1, Quantum Instruments Inc., Hauppauge, NY, USA. An opaque 30.5 x 21.6 cm rectangular divider was placed in the middle between the two binary choice chambers to prevent light interference between the chambers.

To determine whether or not conspecific eggs were involved in mediating *S*. *vittatum* oviposition behavior, an experiment was conducted with the binary choice chamber bioassay in which we observed how many gravid females landed upon and/or oviposited on a substrate with eggs or no eggs when given the two choices simultaneously. Twenty gravid flies were aspirated into the binary choice chamber and exposed to the oviposition substrates for 20 minutes. In each experimental run, 1.0 mL of deionized water was applied to each oviposition substrate halfway (10 m) through the experiment to re-wet the filter paper. As soon as a fly began to oviposit, it was immediately aspirated from the bioassay chamber to reduce potential contamination resulting from the addition of fresh eggs to either substrate. The experiment was replicated ten times; thus a total of 200 individual gravid *S*. *vittatum* were examined. The oviposition response was assessed by recording the number of flies ovipositing on each substrate during the experiment, while the landing response was assessed by recording the number of flies on the substrates at each minute. A Wilcoxon matched-pairs signed rank test was used to compare the means of ovipositing flies on a substrate with eggs and without eggs in the binary choice chamber. The mean number of flies located on the substrate with eggs or no eggs in the binary choice chamber was compared using a paired t-test.

The orientation double chamber bioassay (Fig. 2, Panel b) was used to observe the behavior of gravid *S*. *vittatum* when presented with a substrate with eggs or without eggs. The orientation double chamber consisted of 2 chambers, with a neutral chamber resting on top of a test chamber that in turn rested on a base. The neutral and test chambers were modified 20.3 x 20.3 x 20.3 cm collapsible insect cages (Bioquip, Rancho Dominguez, CA, USA). Black plastic was used to cover the sides, back, and top, leaving the front side covered with clear plastic to allow for observation of fly behavior. The bottom of the neutral chamber was open in order to give the flies the opportunity to migrate into the test chamber. A wooden baffle covered the left half of the opening between the neutral and test chambers, so that flies could be introduced into the neutral chamber without being physically launched into the test chamber. The test chamber was covered with black plastic on the sides and back, and clear plastic on the front. The top of the test chamber was not covered, so that flies could enter from the neutral chamber. The bottom of the test chamber rested directly on top of the base, a 25.4 x 25.4 x 34.3 cm wooden stand with a 5.5 cm hole in its top for holding a 60 x 15 mm Petri dish lid. The Petri dish lid served as the receptacle for the oviposition substrate during the experiment. A 7.5 W incandescent white light (Feit Electric Inc., Pico Rivera, CA, USA) in a standard socket base was centered on the bottom platform of the base, so white light emerging from under the oviposition substrate at the top of the base (7.0± 0.1 foot-candles) served to orient the flies entering the test chamber. In each experimental run, 20 gravid flies were aspirated into the neutral chamber. The test chamber contained either an oviposition substrate with fresh eggs or a control substrate with no eggs. The duration of each run was 20 minutes. Five replicates were conducted, representing observations of 100 individual gravid *S*. *vittatum* for each treatment. To observe whether factors being emitted from the eggs were attracting gravid *S*. *vittatum*, the time it took the flies to migrate from the neutral chamber into the test chamber and land on the oviposition substrate was recorded. The time from landing to oviposition was also recorded.

The Petri dish bioassay (Fig. 2, Panel c) was used to assay the ability of crude extracts, purified compounds and a blend of compounds to induce oviposition of individual gravid flies. These were carried out in inverted 60 x 15 mm glass Petri dishes (Pyrex, Corning Inc., Corning, NY, USA). The dish was lined with a circular layer of cotton wool (U.S. Cotton, Gastonia, NC, USA) with a circular piece of 5.5 cm filter paper (Whatman grade 1, GE Healthcare, Little Chalfont, UK) placed on top to act as the oviposition substrate. Immediately before an experimental run, 40 μL of the control or test stimulus was applied to the filter paper in the Petri dish. The stimulus for the Petri dish bioassay consisted of the test material, the crude extract or individual compounds or solvent only as the control treatment. After the solvent had evaporated, 5.0 mL of deionized water was pipetted onto the treated filter paper. The wet and treated filter paper was then covered with the top of the Petri dish. A single gravid fly was aspirated into the Petri dish at the beginning of the bioassay and the dish immediately covered with a black cloth. The Petri dishes were randomly placed on a wooden light box with a transparent acrylic sheet (2 mm thickness) top. Three fluorescent lights (T8 plant growth, Utilitech, distributed by Good Earth Lighting Inc., Wheeling, IL, USA) inside the light box emitted light equally to the Petri dishes. The mean light intensity measured 1 cm above the acrylic platform in 10 different locations was 115.3 ± 11.6 foot-candles.

Three experiments were conducted using the petri dish bioassay. In the first experiment the test stimuli were methanol or hexane extracts of freshly laid eggs. The control dishes received the solvent only and the experimental dishes received the extracts. Sixty flies were used for each treatment, with the experiment conducted in six runs, each consisting of ten control and ten test dishes. The second experiment evaluated the ability for individual compounds in the non-polar egg extract to stimulate oviposition. Again, a total of 60 flies were used for each treatment, with the experiment run in six batches, with each batch consisting of 10 control and 10 experimental dishes. Finally, the tests of the blended compounds were carried out in two batches, with each batch consisting of 15 control and 15 experimental dishes. Flies were in these experiments exposed to the oviposition substrate impregnated with solvent only (control) or oviposition substrate impregnated with the test compound. Flies in each experimental run were allowed 30 minutes to respond to the stimulus. At the end of each run, the number of flies that oviposited on the substrates was recorded. The data from each run for each treatment were first analyzed to determine if there was homogeneity among the different runs, using a likelihood ratio test. Once this condition was satisfied (i.e. the test for homogeneity did not reject and the data were found to be homogeneous among the runs) the data from the runs were combined and the significance in differences observed in the oviposition probabilities between the control and test datasets was assessed using a Fisher’s exact test.

A blend of the compounds identified in the hexane egg extract was also tested in the second petri dish assay experiment. The artificial blend consisted of pentadecene, hexadecene, *cis*-9-tetradecen-1-ol and tridecene at a ratio of 7.8: 2.3: 2.3: 1.0. This ratio was based upon the relative proportion of the compounds found in the hexane egg extract. Details on the quantification of the amount of each compound present in the egg extracts may be found in S1 Text.

### Electroantennography

A live, restrained, gravid *S*. *vittatum* female was mounted with two glass electrodes filled with 0.1 M KCl solution and a chlorinated silver-silver wire (0.2 mm diameter) inserted into the KCl solution. The indifferent electrode was fixed to the fly by puncturing the scutum, and the recording electrode was connected to the tip of an antenna. After mounting the fly on the electrodes, a steady baseline was achieved on the electroantennograph (EAG) readout before exposing the fly to experimental stimuli. Humidified and charcoal-filtered air (1000 mL/m) was continuously delivered through a metal tube (1 cm i.d.) that was oriented toward the mounted fly. To provide the stimulus to the mounted fly, 10 μL of the stimulus solution prepared at a concentration of 80μM in hexane was applied to filter paper (Whatman, No. 1, GE Healthcare, Little Chalfont, UK). The solvent was given one minute to evaporate, and the filter paper was then inserted into a glass Pasteur pipette (Fisher Scientific, Pittsburgh, Pennsylvania, USA). The prepared glass pipette was connected to a plastic tube, which connected to the stimulus controller (Stimulus Controller CS-55, Syntech, the Netherlands) and the tip of the pipette was inserted into a side hole on the metal tube. Once inserted into the metal tube, a 0.2 s pulse of air generated from a controller carried the stimulus over the mounted fly. The EAG response was collected using a data acquisition controller (IDAC-4, Syntech, the Netherlands) and analyzed using the EAG 2000 software (Syntech, the Netherlands) on a laptop computer. A single gravid fly was used for each recording session. Each recording session was initiated with a mechanical control (air) stimulus, solvent control (hexane) stimulus, followed by three rounds of exposure to randomly assorted test stimuli. Before and after each round the fly was exposed to1-octen-3-ol, which served both as a positive control and a reference standard. Previous studies had shown that this compound elicited a strong response in the EAG when used to stimulate *S*. *vittatum* (data not shown). Each recording session was concluded with repeats of the solvent alone and mechanical control stimuli. The response values for the test stimuli were normalized to the fly’s response to 1-octen-3-ol (100%), thus accounting for fly-to-fly variation in the intensity of the depolarization. The normalized data from each test stimulus was compared to the normalized control data by a one-way analysis of variance (ANOVA) followed by Fisher’s Least Significant Difference (LSD) multiple comparison test.

## Results

**Fig 3:**
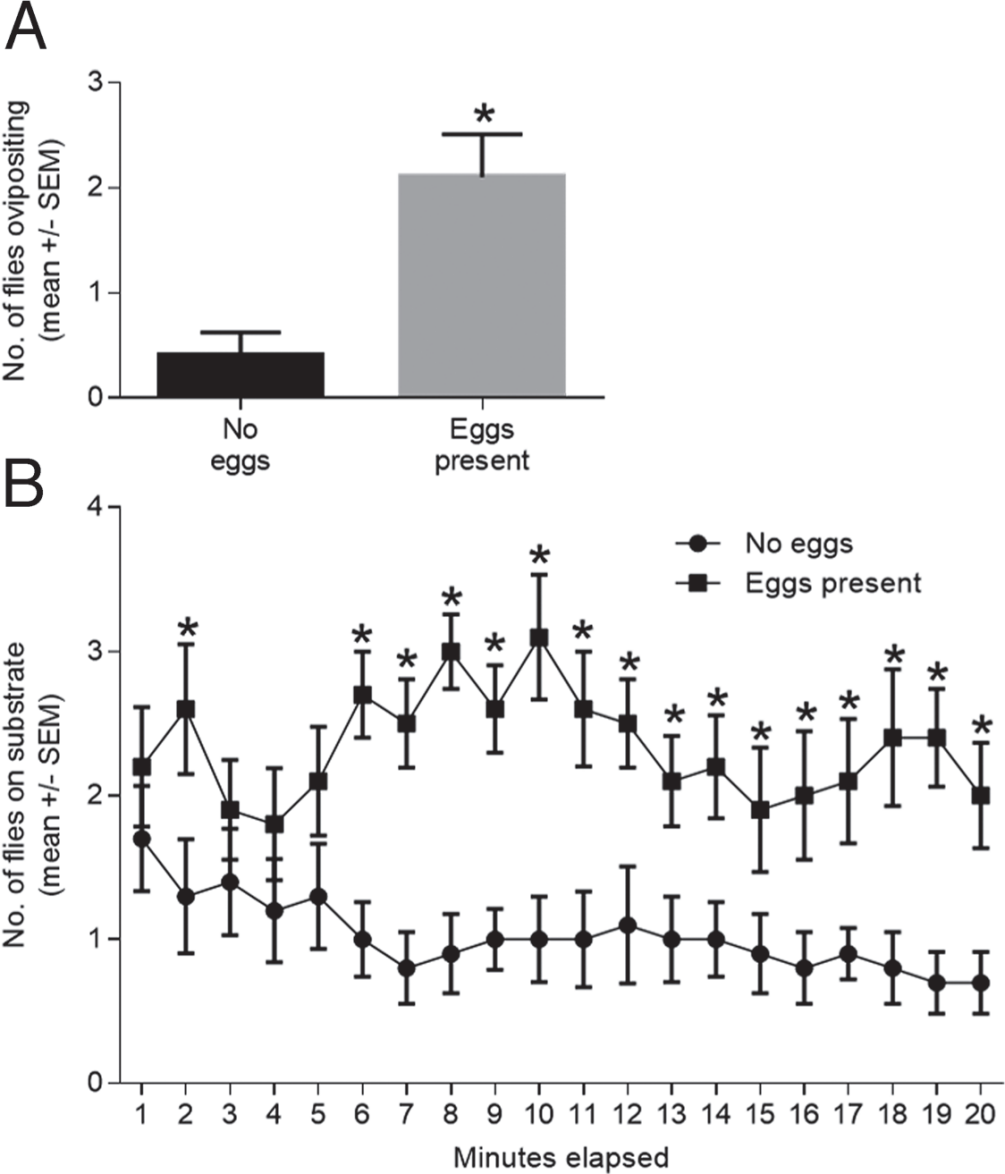
The behavioral responses of gravid *S*. *vittatum* to fresh conspecific eggs in the binary choice chamber assay. Panel A: Oviposition preference of gravid *S*. *vittatum* on a substrates containing or lacking eggs. Values significantly different from the control are denoted by an asterisk [*] (Wilcoxon matched-pairs signed rank test, *P* = 0.002). Panel B: Orientation response represented the number of flies located after landing on a substrate with eggs or lacking eggs. Values significantly different from the control are denoted by an asterisk [*] (t-test, *P* < 0.05).

Gravid *S*. *vittatum* preferred to oviposit more on an oviposition substrate containing fresh eggs than the control substrate in the binary choice chamber bioassay (Fig. 3, Panel A; *P* = 0.002). More gravid *S*. *vittatum* were located on the substrate with eggs than on the substrate with no eggs (Fig. 3, Panel B; *P* < 0.05). However, the flies did not consistently begin to show a preference for the substrate containing the eggs until 5 minutes after the experimental run was initiated. During the first 5 minutes of an experimental run, frenetic and random movement by the flies was noted, perhaps resulting from the disturbance associated with being introduced into the chamber.

**Fig 4:**
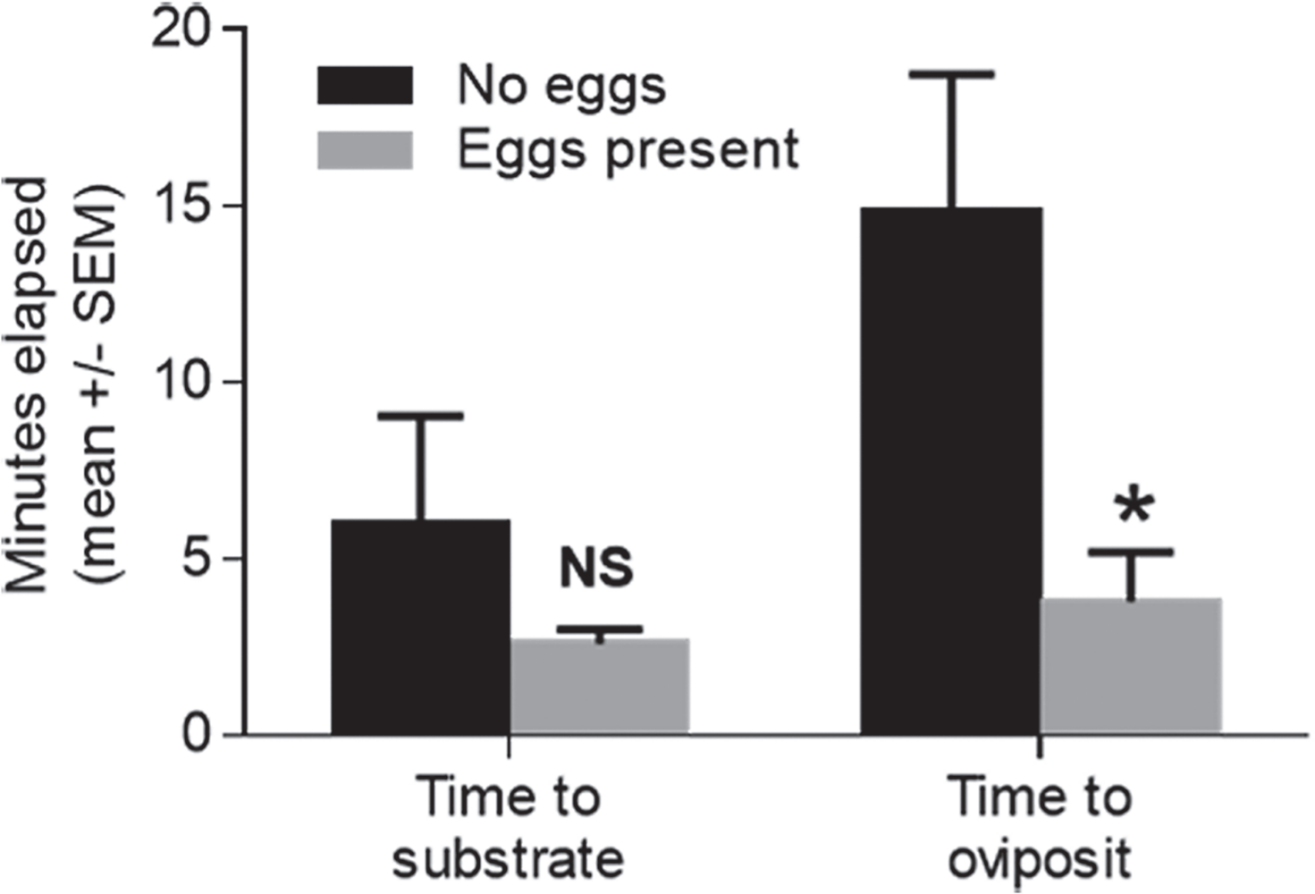
The behavioral responses of gravid *S*. *vittatum* to fresh conspecific eggs in the orientation double chamber assay. Assays were conducted in the presence or absence of fresh eggs, in the lower chamber, as described in Materials and Methods. Values that were significantly different from the control are indicated by an asterisk [*] (unpaired t-test, *P* < 0.05) and those which were not significantly different values are indicated by NS (*P* > 0.05).

**Fig 5:**
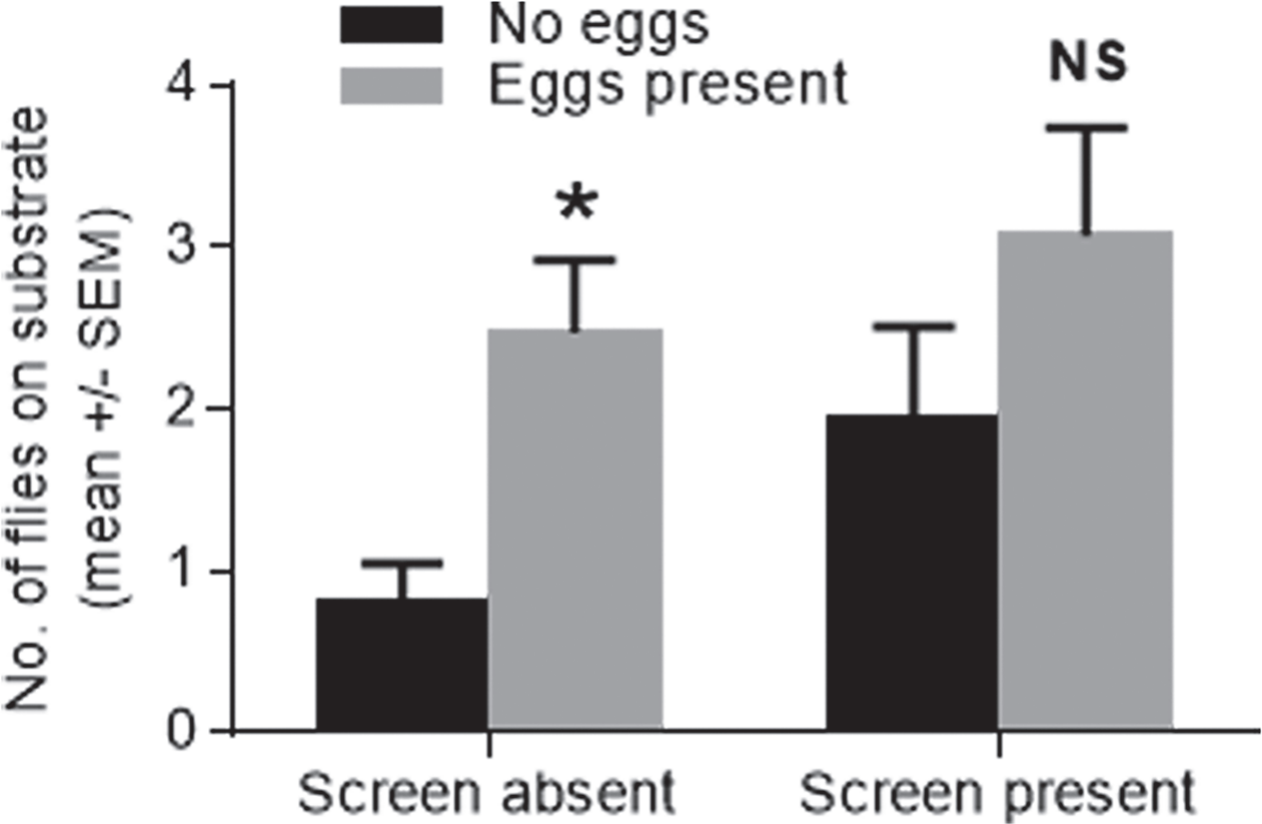
The behavioral responses of gravid *S*. *vittatum* to uncovered or covered fresh conspecific eggs. Values represent the number of flies present on the substrate or mesh screen after 20 minutes. Significantly different values are indicated by an asterisk [*] (unpaired t-test, *P* < 0.05) and values that were not significantly different are indicated by NS (*P* > 0.05).

As a first step in addressing the nature of the attractive response to the fresh eggs, the orientation double chamber bioassay was developed in which flies were introduced into an neutral chamber and then timed as to how long it took the files to move into an adjacent test chamber containing either a substrate with eggs or a control substrate without eggs. In addition, the time to oviposition once in the test chamber and in contact with the substrate was also measured. The time it took for flies to move from the neural chamber into the test chamber containing fresh eggs was not significantly different when the test chamber contained fresh eggs or water soaked substrate alone (Fig. 4; *t* = 1.105, *P* = 0.300). However, once flies had landed on the oviposition substrate, they oviposited significantly more quickly on the substrate with eggs than did the flies that landed on the substrate with no eggs (Fig. 4; *t* = 2.646, *P* = 0.0294). This difference was statistically significant only when the substrates were not covered by a wire mesh, preventing direct fly-egg contact (Fig. 5; *t* = 3.283, *P* = 0.0028).

**Fig 6:**
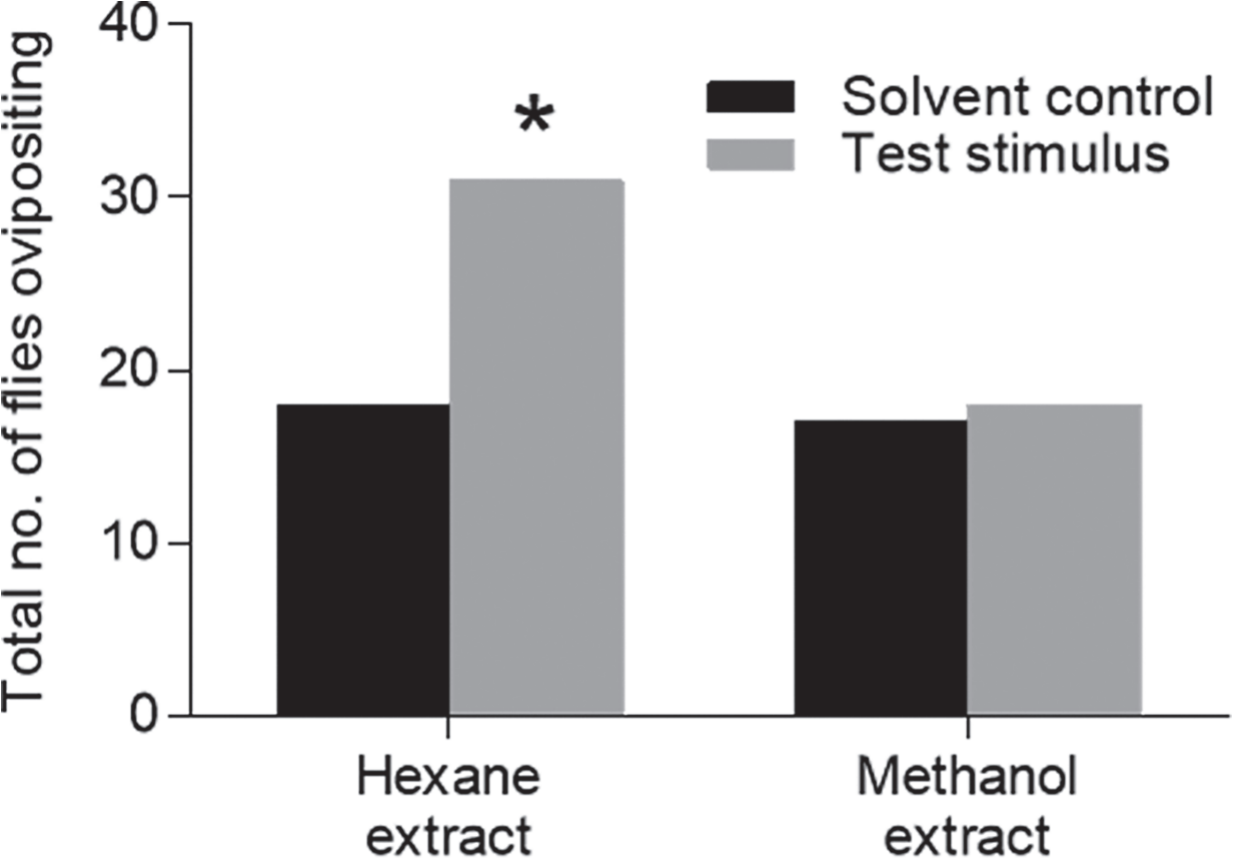
Oviposition responses of gravid *S*. *vittatum* egg extracts in the Petri dish assay. The response values of the crude extracts were compared to their respective solvent controls. A significant difference between the solvent control and the extract is indicated by an asterisk [*] (Fisher’s exact test, P < 0.05). Each column represents an assay run on 60 individual flies.

To investigate the pheromones present in the eggs that induced the responses seen in the behavioral assays, crude extracts of eggs were prepared in polar (methanol) and non-polar (hexane) solvents and tested for activity in the Petri dish assay. Crude hexane extracts from eggs induced significantly more oviposition in the Petri dish assay than did controls containing the solvent alone (Fig. 6, Fisher’s exact test, *P* < 0.05). In contrast, methanol extracts of fresh eggs elicited no more oviposition in the Petri dish assay than did substrates containing methanol alone (Fig. 6).

**Fig 7:**
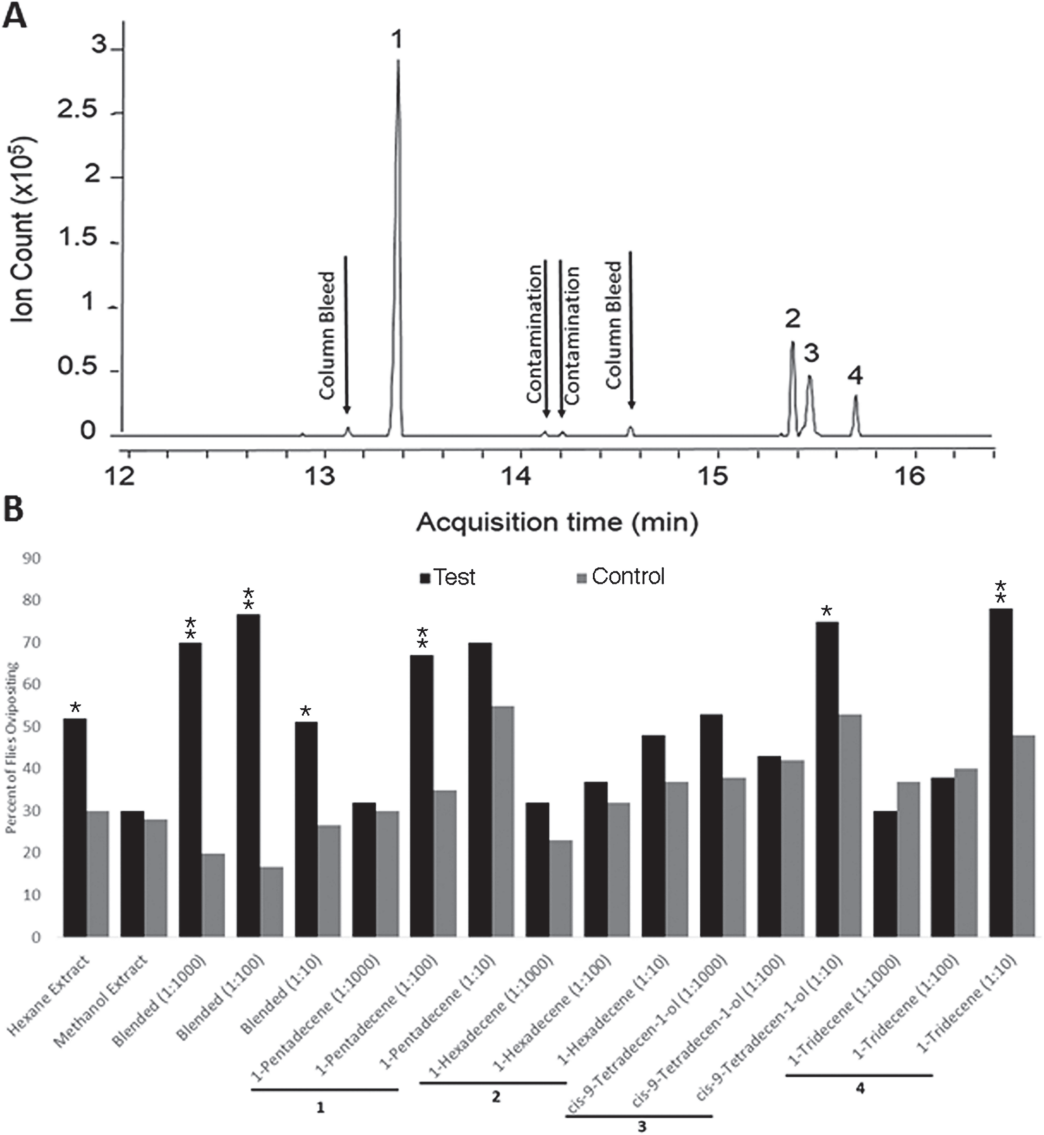
Isolation and characterization of compounds present in hexane crude extracts of *S*. *vittatum* eggs. Panel A: Gas chromatographic analysis of hexane crude extracts. Numbers highlight the major peaks. The minor peaks were identified in blank runs as well as in the egg extract and were therefore attributed to contamination. Panel B: Oviposition response in the Petri dish assay of gravid *S*. *vittatum* to the individual compounds identified in the hexane crude extracts (1-hexadecene, 1-pentadecene, 1-tridecene, and *cis*-9-tetradecen-1-ol). Numbers under the x- axis correspond to the peaks shown in Panel A. Compounds were tested at 3 different dilutions (1:1000, 1:100, 1:10 w/v). Response values of the individual compounds that were significantly different than the solvent control are indicated by a single asterisk [*](Fisher’s exact test, *P* < 0.05) or a double asterisk [**](Fisher’s exact test, *P* < 0.005). Because different numbers of flies were used in the tests of the individual compounds and the blends, the data are presented as the percentage of flies ovipositing on the substrate. Blend = a mixture of pentadecene, hexadecene, *cis*-9-tetradecen-1-ol and tridecene at a ratio of 7.8: 2.3: 2.3: 1.0 (the ratio found in the hexane extract; see S1 Text).

The compounds present in the crude hexane extract were then separated by gas chromatography. Four abundant peaks were identified in the hexane extracts (Fig. 7, Panel A), which were then identified as 1-pentadecene, 1-hexadecene, *cis*-9-tetradecen-1-ol, and 1-tridecene by mass spectroscopy. Three additional minor peaks were seen (Fig. 7, Panel A). These were also present in blank samples run in parallel, indicating that they were contaminants from the column itself. Three of these compounds corresponding to the major peaks were commercially available, but *cis*-9-tetradecen-1-ol was not. The latter compound was therefore synthesized *de novo* for additional experiments. Details of the synthesis of this compound are provided in S1 Text and the synthetic scheme is summarized in Fig. 1. The four compounds identified in the crude extract were tested at three different dilutions (1:1000, 1:100, and 1:10 w/v) for their ability to induce oviposition in the Petri dish bioassay. Three of the compounds identified in the crude extract (1-pentadecene, 1-tridecene, and *cis*-9-tetradecen-1-ol) induced a significantly greater degree of oviposition than did the solvent alone (Fisher’s exact test, Fig. 7, Panel B). A blend of all compounds (in the natural ratio of 7.8: 2.3: 2.3: 1.0 for pentadecene, hexadecene, *cis*-9-tetradecen-1-ol and tridecene respectively) was active at a lower dilution than were any of the individual compounds alone (Fig. 7, Panel B).

**Fig 8:**
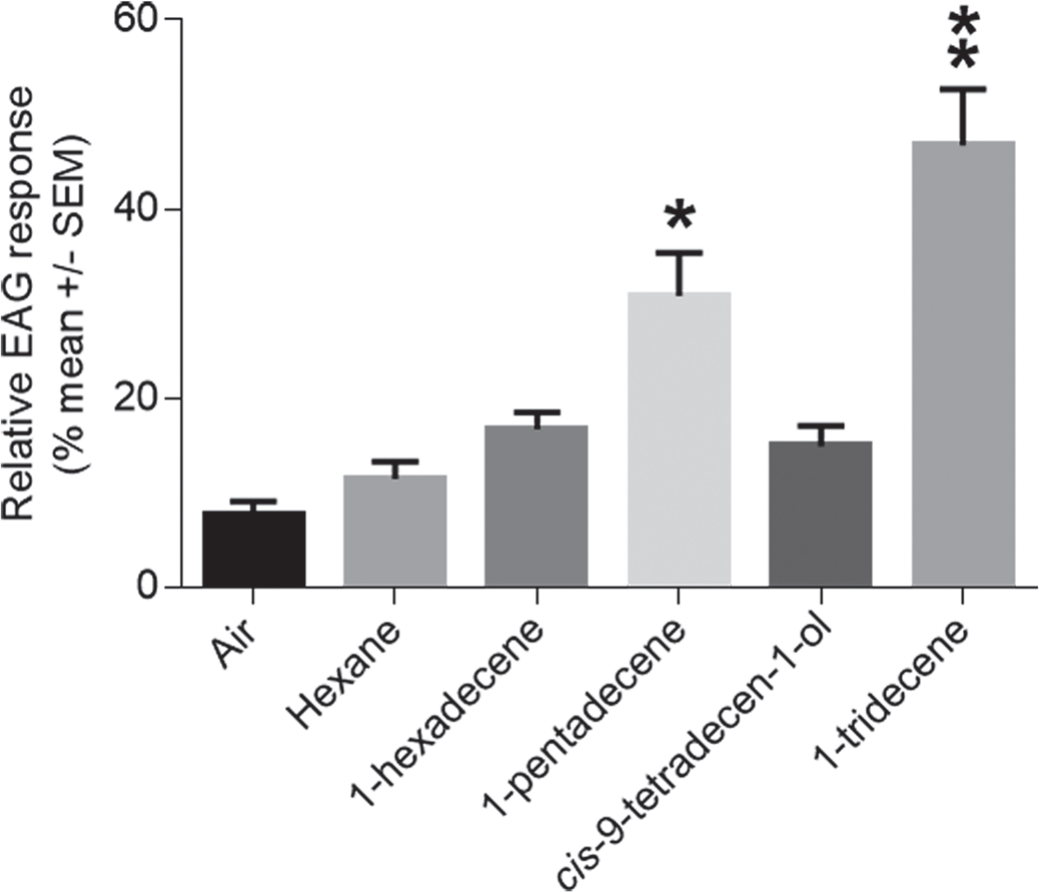
EAG responses of gravid *S*. *vittatum* to individual compounds identified from the crude extract of conspecific eggs. Assays were carried out on a live restrained fly as described in Materials and Methods. Responses that are significantly different from the solvent control are denoted by the asterisks * and ** (Fisher’s LSD, *P* < 0.005 and *P* < 0.0001, respectively).

Electroantennogram experiments were then conducted to assess the possible olfactory response to the individual compounds found in the hexane extract. Two of the three compounds active in the Petri dish assay (1-pentadecene and 1-tridecene) elicited a significantly higher response in the EAG than the solvent control of hexane (Fig. 8; *P* < 0.005), while *cis*-9-tetradecen-1-ol (the third active compound in the Petri dish assay) and 1-hexadecene (which was not active in the Petri dish assay) did not elicit an EAG response.

## Discussion

Gravid colonized *S*. *vittatum* prefer to oviposit on a substrate with fresh eggs in comparison to a substrate without eggs, which coincides with behavioral experiments assessing the communal oviposition phenomenon with the *S*. *damnosum s*.*l*. and *S*. *ochraceum s*.*l*. species complexes [12,16]. Our findings demonstrate that communal oviposition behavior is likely conserved among species in the genus *Simulium* and the colonized *S*. *vittatum* females used here represented a suitable model for further investigating the factors associated with black fly communal oviposition.

In the experiments conducted in the orientation double chamber, flies tended to enter the test chamber more quickly when fresh eggs were present, although the differences in time failed to reach statistical significance. These studies may have been confounded to some extent by the relative positioning of the neutral and test chamber, with the test chamber being located below the entry chamber. It is possible that the flies initially exhibit a negative geotropism, which based on the apparatus design would have reduced their overall propensity to enter the test chamber. For example, Davies noted that gravid females of several species in the *S*. *damnosum* complex assembled initially on the tops of flowering plants near their oviposition site before moving down to trailing vegetation to oviposit at dusk [17]. However, once entering the test chamber, flies oviposited significantly more quickly when eggs were present. Furthermore, when the eggs were covered by a fine wire mesh, the difference in oviposition seen between substrates containing eggs and lacking eggs was no longer statistically significant. These data suggest that the eggs likely exhibit some attraction to flies over a short distance but once in contact with the eggs, the gravid flies were induced to remain on site and oviposit. Taken together, these data suggest that the communal oviposition response may involve both tactile and olfactory stimuli.

**Fig 9:**
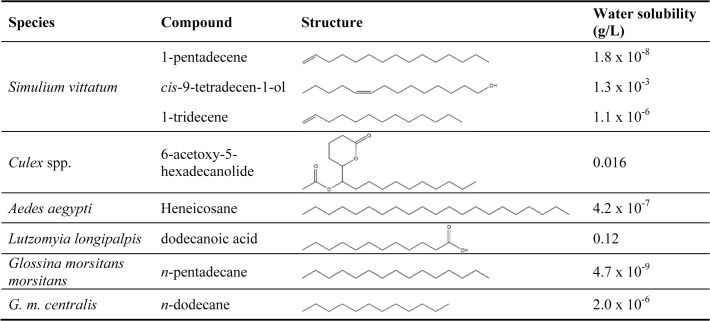
Compounds identified as oviposition pheromones in Dipteran species. Oviposition pheromones were identified in the following sources: *Simulium vittatum*, this work; *Culex spp*. [14]; *Aedes aegypti* [19]; *Lutzomyia longipalpis* [5]; *Glossina morsitans morsitans* and *G*. *m*. *centralis* [20].

Chemical fractionation of the crude extract from *S*. *vittatum* fresh eggs coupled with the dish bioassay allowed us to identify the active compounds inducing oviposition as 1-pentadecene, 1-tridecene, and *cis*-9-tetradecen-1-ol. Intriguingly, the characteristic features of these compounds are similar to other Dipteran oviposition pheromones (Fig. 9), with some similarities occurring throughout the Order. Dipteran evolution has occurred over a 260 million year period and is characterized by periodic radiations resulting in several relatively diverse taxonomic groups [18]. As seen in Fig. 9, relatively low molecular weight aliphatic compounds (fatty acids, alcohols) appear to have been favored for selection as oviposition pheromones several times during evolution, with examples occurring both within the oldest families—Culicidae, Simuliidae, Psychodidae—as well as the more recently evolved families, e.g. Glossinidae (tsetse flies). The evolutionary period between the origin of the two groups conservatively represents a time span of 125–150 million years [18].

The *S*. *vittatum* communal oviposition pheromone has also been recorded as an active agent in other taxonomic orders. For example, 1-pentadecene has been demonstrated to be a potential aggregation pheromone in the beetle *Tribolium confusum* (Coleoptera: Tenebrionidae) [21]. *Parastizopus transgariepinus*, another tenebrionid beetle, uses 1-tridecene as a male-produced sex pheromone [22]. Moreover *cis*-9-tetradecen-1-ol acetate was identified as a pheromone in two Lepidopterans, the southern armyworm, *Spodotera* (= *Prodenia) eridania*, and the leaf roller *Argyrotaenia ljungianai* [23,24].

Commercially available preparations of three of the compounds identified in the hexane extracts were used in the petri dish assays to assess their ability to induce oviposition. Neither of the two commercial compounds found to be active in the petri dish assay (1-pentadecene and 1-tridecene) were 100% pure. It is therefore formally possible that the contaminants in the commercial preparations could have been responsible for eliciting oviposition in the petri dish assay, while the major constituent in the commercial preparations was inactive. This possibility to be highly unlikely as the compounds were found to be the major constituents of the hexane extracts from freshly laid eggs. It is unlikely that the contaminants in the commercial preparations were also present in undetectable amounts in the egg extracts, yet still present in sufficient amounts to elicit the oviposition chamber. Taken together, this suggests that the major compounds identified in the egg extracts were indeed responsible for eliciting the oviposition response from gravid flies.

Two of the three compounds identified as inducing oviposition in the Petri dish assay also induced a response in the EAG, while the third compound active in the petri dish assay did not (*cis*-9-tetradecen-1-ol) did not. This suggests that the oviposition behaviors induced by fresh eggs may be mediated through a combination of olfactory and chemotactile receptors, as any response to chemotactile receptors would likely not have been detected in the EAG. This hypothesis is supported by the fact that the experiments with the mesh covered eggs showed that oviposition behavior was enhanced when eggs were both present and accessible rather than only being present. This hypothesis is supported by reports of oviposition behavior of *Simulium* spp. females seen in nature. For example, Golini and Davies [8] observed a tactile investigative behavior by black flies at an oviposition site. Flies would land on a potential oviposition substrate, tap their forelegs around the surface, and if they found it to be unsuitable they would then fly away. Black flies contain an array of sensory receptors on their fore-tarsi [25] and this behavior could represent the use of tactile chemoreceptors to determine whether an oviposition substrate is suitable. In this context, it is interesting to note that all three of the oviposition-inducing compounds identified here are relatively insoluble in water (Fig. 9) meaning that they would likely adhere firmly to the lipophilic surface of the black fly egg in the fast running water in which the flies lay their eggs under natural conditions and therefore would not be easily washed away. Among other Diptera, tarsal “taste” sensilla respond to non-volatile lipophilic compounds to modulate different types of behavior patterns, e.g., mating behavior in *Drosophila* spp. Additionally, a lipophilic ligand-binding protein has been identified in both the taste (labella, tarsi) and olfactory (antenna) organs of the blow fly, *Phormia regina*, thereby providing a common molecular mechanism for both senses [26].

While the studies described here have concentrated on chemical factors that induce oviposition, it is clear that many other factors influence the selection of an oviposition site by gravid black flies, including air current over water, light level, substrate color and reflectance, and texture of the substrate [6–8,27]. These natural factors are not easily replicated in laboratory experiments, but the successful identification of the oviposition inducing compounds in egg extracts opens the way to using these compounds as attractants in field studies where the other necessary environmental cues are present. Further, although the blend tested here reflected the proportions of the compounds present in the hexane extract, optimizing the composition of the blend of the active compounds would be a major priority for the eventual development of such a blend for use as bait in a trap to collect gravid black flies.

It has been suggested that identification and synthesis of a black fly oviposition pheromone could lead to an attractant for the capture of gravid females [10]. The use of oviposition pheromones such as these described here could greatly increase the number of *Simulium* eggs deposited on an attractive substrate [27] and when combined with a suitable ovicidal chemical could serve as a possible control method for *Simulium* species, including those medically important species that serve as the vector for the causative agent of river blindness, the parasite *Onchocerca volvulus*.

## Supporting Information

S1 Text Detailed methods for synthesis of *cis*-9-tetradecen-1-ol and determination of ratio of identified compounds in the hexane extract. (DOCX)

## References

[1] Prokopy RJ, Roitberg BD (2001) Joining and avoidance behavior in nonsocial insects. Annual Review of Entomology 46: 631–665. 11112182 10.1146/annurev.ento.46.1.631

[2] Kahoro H, Odongo H, Saini RK, Hassanali A, Rai MM (1997) Identification of Components of the Oviposition Aggregation Pheromone of the Gregarious Desert Locust, *Schistocerca gregaria* (Forskal). Journal of Insect Physiology 43: 83–87. 12769932 10.1016/s0022-1910(96)00051-0

[3] McCall PJ, Cameron MM (1995) Oviposition pheromones in insect vectors. Parasitol Today 11: 352–355. 15275321 10.1016/0169-4758(95)80192-8

[4] Dumesnil JG, Laurenceau JL, Shoucri RM (1982) [Evaluation of function in left ventricle hypertrophy by echocardiography]. Arch Mal Coeur Vaiss 75: 1149–1158. 6219647

[5] Dougherty M, Hamilton G (1997) Dodecanoic acid is the oviposition pheromone of *Lutzomyia longipalpis* . Journal of Chemical Ecology 23: 2657–2671.

[6] Coupland JB (1991) Oviposition response of *Simulium reptans* (Diptera: Simuliidae) to the presence of conspecific eggs. Ecological Entomology 16: 11–15.

[7] Coupland JB (1992) Effect of egg mass age on subsequent oviposition by *Simulium reptans* (Diptera: Simuliidae). Journal of Medical Entomology 29: 293–295. 1495045 10.1093/jmedent/29.2.293

[8] Golini VI, Davies DM (1975) Relative response to colored substrates by ovipositing blackflies (Diptera: Simuliidae). I. Oviposition by *Simulium* (Simulium) *verecundum* Stone and Jamnback. Canadian Journal of Zoology 53: 521–535. 1131749 10.1139/z75-067

[9] Muirhead-Thompson RC (1956) Communal oviposition in *Simulium damnosum* Theobald (Diptera, Simuliidae). Nature 178: 1279–1299.

[10] McCall PJ, Heath RR, Dueben BD, Wilson MD (1997) Oviposition pheromone in the *Simulium damnosum* complex: biological activity of chemical fractions from gravid ovaries. Physiological Entomology 22: 224–230.

[11] McCall PJ (1995) Oviposition aggregation pheromone in the *Simulium damnosum* complex. Medical and Veterinary Entomology 9: 101–108. 7787216 10.1111/j.1365-2915.1995.tb00165.x

[12] McCall PJ, Trees AJ, Walsh JF, Molyneux DH (1994) Aggregated oviposition in the *Simulium damnosum* complex is mediated by eggs in a laboratory bioassay. Medical and Veterinary Entomology 8: 76–80. 8161850 10.1111/j.1365-2915.1994.tb00390.x

[13] Gray EWN R (1999) Large scale laboratory rearing of black flies In: Maramorosch KM, F., editor. Maintenance of human, animal and plant pathogen vectors. New Delhi, India: Oxford and IBH Publishing Co pp. 85–100.

[14] Laurence BR, Mori K., Otsuka T., Pickett J. A., and Wadhams L. J. (1985) Absolute configuration of mosquito oviposition attractant pheromone, 6-acetoxy-5-hexandecanolide. Journal of Chemical Ecology 11: 643–648. 10.1007/BF00988573 24310128

[15] Osgood CE (1971) An oviposition pheromone associated with the egg rafts of Culex tarsalis. Journal of Economic Entomology 64: 1038–1041. 5122321 10.1093/jee/64.5.1038

[16] Rodriguez-Perez MA, Valdivieso-Lopez NL, McCall PJ (2003) Aggregated oviposition in *Simulium ochraceum s*.*l*. (Diptera: Simuliidae), an important neotropical vector of *Onchocerca volvulus* . Annals of Tropical Medicine and Parasitology 97: 203–207. 12803877 10.1179/000349803235001534

[17] Davies JB (1962) Egg-laying habits of *Simulium damnosum* Theobald and *Simulium medusaeforme* form hargrevesi Gibbins in northern Nigeria. Nature 196: 149–150.

[18] Wiegmann BM, Trautwein MD, Winkler IS, Barr NB, Kim JW, et al (2011) Episodic radiations in the fly tree of life. Proceedings of the National Academy of Sciences of the United States of America 108: 5690–5695. 10.1073/pnas.1012675108 21402926 PMC3078341

[19] Seenivasagan T, Sharma KR, Sekhar K, Ganesan K, Prakash S, et al (2009) Electroantennogram, flight orientation, and oviposition responses of *Aedes aegypti* to the oviposition pheromone n-heneicosane. Parasitology Research 104: 827–833. 10.1007/s00436-008-1263-2 19018567

[20] Saini RK, Hassanali A, Andoke J, Ahuya P, Ouma WP (1996) Identification of major components of larviposition pheromone from larvae of tsetse flies *Glossina morsitans morsitans* Westwood and *Glossina morsitans centralis* Machado. Journal of Chemical Ecology 22: 1211–1220. 10.1007/BF02266961 24226080

[21] Verheggen F, Ryne C, Olsson PO, Arnaud L, Lognay G, et al (2007) Electrophysiological and behavioral activity of secondary metabolites in the confused flour beetle, *Tribolium confusum* . Journal of Chemical Ecology 33: 525–539. 17265176 10.1007/s10886-006-9236-3

[22] Geiselhardt S, Ockenfels P, Peschke K (2008) 1-Tridecene—male-produced sex pheromone of the tenebrionid beetle Parastizopus transgariepinus. Naturwissenschaften 95: 247–251. 17898976 10.1007/s00114-007-0312-5

[23] Jacobson M, Redfern RE, Jones WA, Aldridge MH (1970) Sex pheromones of the Southern Armyworm Moth: Isolation, identification, and synthesis. Science 170: 542–544. 5507205 10.1126/science.170.3957.542

[24] Velcheva N, Pjatnova Y, Kislicina T, Voinova V, Vendilo N (2012) Evaluation of pheromone Lures for monitoring *Argyrotaenia ljungiana* (Thunberg 1797) (Lepidoptera, Tortricidae). Rastenievudni Nauki 49: 15–22.

[25] Sutcliffe JF, McIver SB (1976) External morphology of sensilla on the legs of selected black fly species (Diptera: Simuliidae). Canadian Journal of Zoology 54: 1779–1787. 974936 10.1139/z76-207

[26] Ozaki M, Morisaki K, Idei W, Ozaki K, Tokunaga F (1995) A Putative Lipophilic Stimulant Carrier Protein Commonly Found in the Taste and Olfactory Systems—a Unique Member of the Pheromone-Binding Protein Superfamily. European Journal of Biochemistry 230: 298–308. 7601113 10.1111/j.1432-1033.1995.0298i.x

[27] Bellec C (1976) Captures d'adultes de *Simulium damnosum* Theobald, 1903 (Dipetra, Simuliidae) a l'aide de plaques d'aluminium, en Afrique de l'Quest. Medical Parisitology and Entomology 14: 209–217.

