# Supplementary material for: Identification of Communal Oviposition Pheromones from the Black Fly Simulium vittatum
Source: PLoS One. 2015 Mar 18;10(3):e0118904. doi: 10.1371/journal.pone.0118904 (PMC4364695; doi:10.1371/journal.pone.0118904)
Supplement: S1 Text — (DOCX) [file pone.0118904.s001.docx]

**S1 text**

**Detailed methods for synthesis of *cis*-9-tetradecen-1-ol and determination of ratio of identified compounds in the hexane extract.**

**Synthesis of cis-9-Tetradecen-1-ol**

General Synthesis Methods

Reactions where exclusion of water was necessary were performed using glassware dried in a 150 °C oven overnight, then flame-dried with a butane torch and flushed with dry argon to remove residual air and moisture. All reactions were performed under an atmosphere of dry Argon. Immediately prior to use solvents were dried using Pure Solv^TM^ solvent purification system, or in the case of pyridine, freshly distilled from sodium hydroxide under an inert atmosphere. Dried solvents were stored over 4Å molecular sieves. Anhydrous DMF was purchased from Sigma Aldrich (Milwaukee, WI, USA).

Powder X-ray Diffraction (PXRD) measurements were collected at room temperature on a Bruker AXS D8 Advance instrument at 50 kV and 40 mA for Cu Kα (λ = 1.5418 Å) with a scan speed of 1 degree/min and a step size of 0.02 degree in 2θ at room temperature. Infrared spectroscopy was measured using an Agilent Cary 630 FTIR coupled to a diamond ATR. All samples were loaded onto the crystal as a thin film and recorded as transmittance from 400-4000 cm^-1^. Molecular ions were detected on an Agilent 7200 QToF equipped with an electron ionization (EI) source or an Agilent 6540 QToF equipped with an electron spray ionization (ESI) source. NMR spectra were collected on a Varian Direct Drive 500 Hz NMR spectrometer coupled with a cryo probe using standard pulse sequences. Chemical shifts are reported in ppm, referenced to residual solvent resonances (CDCl_3_ δ_H_ 7.25, δ_C_ 77.0 ppm). All coupling constants are measured in Hz.

Synthesis of Argentous Oxide

An aqueous solution of sodium hydroxide (1.75 g, 43.8 mmol in 50 mL) was heated to 85 °C, and added to an aqueous solution of silver nitrate (7.5 g, 44.4 mmol in 50 mL) also heated to 85 °C, resulting in a brown precipitate. The solid was filtered and washed with hot water (50 mL), 95% ethanol (50 mL) and finally absolute ethanol (50 mL). The precipitate was dried in a vacuum oven at 150 °C at -30 mmHg overnight yielding a brown micro-crystalline solid (4.57g, 90%). IR (solid) ν: 952, 651, 530 cm^-1^, XRPD (2θ): 32, 38, 55, 65, 68, 77 °, which was comparable to literature values [[1](#_ENREF_1)].

Synthesis of 8-(benzyloxy)octan-1-ol (**2**)

A mixture of 1,8-octanediol **1** (1.053 g, 7.2 mmol) and freshly prepared Ag_2_O (2.503 g, 10.8 mmol, 1.5 eq) were suspended in dichloromethane (60 mL). Benzyl bromide (942 μL, 7.92 mmol, 1.1 eq) was added to the solution and allowed to stir at ambient temperature for 15 h in a vessel covered in aluminum foil to minimize the photodecomposition of silver(I) oxide. The suspension was filtered and the solvent removed under reduced pressure. The mono-protected product was purified via NP-MPLC (100 % hexane to 100% EtOAc over 15 min, isocratic EtOAc which was held for 5 min on silica) to afford a colorless oil (1.607 g, 6.8 mmol, 94% yield). IR (film) ν: 3312 (br), 3059, 2933, 2585, 1640, 1458, 1097, 702 cm^-1^. ^1^H NMR (500 MHz, CDCl_3_): δ 7.34 (m, 2H), 7.33 (m, 3H), 4.50 (s, 2H), 3.63 (t, *J*=6.6, 2H), 3.46 (t, *J*=6.6, 2H), 1.64-1.46 (m, 8H), 1.41-1.27 (m, 4H) ppm. ^13^C NMR (125 MHz, CDCl_3_): δ 138.7, 128.3 (2C), 127.6 (2C), 127.5, 72.9, 70.5, 63.0 32.7, 29.7, 29.4, 29.4, 26.1, 25.7 ppm. EIMS *m/z* (%): 236.1792 (31, [M]^+^; calc’d 236.1776 for C_15_H_24_O_2_), 108.0543 (71), 107.0644 (97), 92.0670 (82), 91.0695 (100), 79.0572 (59), 67.0575 (64), 55.0573 (55); ESI HRMS [M + H]^+^ calc’d for C_15_H_25_O_2_: 237.1855; found: 237.1859.

Synthesis of 8-(benzyloxy)octyl-4-methylbenzenesulfonate (**3**)

Powdered tosyl chloride (890 mg, 4.66 mmol, 1.1 eq) was added portion wise to a stirred ice-cooled solution of **2** (1.00 g, 4.21 mmol) in anhydrous pyridine (3.9 mL). The mixture was allowed to stand at 4 °C overnight under an inert atmosphere. The reaction was quenched with water (5 mL) and extracted with diethyl ether (5 mL). The organic layer was sequentially washed with dilute HCl, deionized water, saturated NaHCO_3_ and finally once again with deionized water. The organic layer was dried over MgSO_4_, filtered and the solvent removed *in vacuo*. The final product was purified via NP-MPLC (100 % hexane to 40% EtOAc over 10 min. The gradient was then ramped to 100% EtOAc which was held for 5 min on silica) affording a yellow oil (1.48 g, 3.80 mmol, 90% yield). IR (film) ν: 3033, 2929, 2858, 1361, 1179, 1100, 1015, 1037, 668 cm^-1^. ^1^H NMR (500 MHz, CDCl_3_): δ 7.78 (d, *J*=8.3, 2H), 7.34 (d, *J*=8.5, 2H), 7.34 (m, 2H), 7.33 (m, 3H), 4.49 (s, 2H), 4.00 (t, *J*=6.36, 2H), 3.44 (t, *J*=6.36, 2H), 2.45 (s, 3H), 1.65-1.54 (m, 4H), 1.35-1.16 (m, 8H) ppm. ^13^C NMR (125 MHz, CDCl_3_): δ 144.7, 144.6, 138.6, 129.8 (2C), 129.8 (2C), 128.3, 127.9 (4C), 72.9, 70.7, 70.6, 29.7, 28.9, 28.8, 28.7, 28.7, 26.0, 25.2 ppm. EIMS *m/z* (%):207.0298 (44), 173.0258 (29), 107.0482 (31), 92.0595 (17), 91.0537 (100), 81.0692 (15), 69.0694 (15), 67.0541 (21); ESI HRMS [M + H]^+^ calc’d for C_22_H_31_O_4_S: 391.1943; found: 391.1968.

Synthesis of (((8-iodooctyl)oxy)methyl)benzene (**4**)

Solid NaI (1.43 mg, 9.56 mmol, 2.6 eq) was added to a stirred solution of **3** (1.44 mg, 3.68 mmol) in DMF (4 mL). The solution was heated to 50 °C and allowed to stir for 4 h, where the reaction was diluted with water and extracted with hexane. The hexane was washed with water and brine, dried over MgSO_4_ and the filtered solvent removed under reduce pressure. The resulting was oil purified by NP-MPLC (100 % hexane to 45% EtOAc over 15 min, the gradient was ramped to 100% EtOAc which was held for 5 min on silica), yielding a yellow oil (650 mg, 1.88 mmol, 51% yield). IR (film) ν: 3029, 2929, 2854, 1458, 1365, 1100, 736, 702, 506 cm^-1^. ^1^H NMR (500 MHz, CDCl_3_): δ 7.34 (d, *J*=8.5, 2H), 7.34 (m, 2H), 7.33 (m, 1H), 4.51 (s, 2H), 3.47 (t, *J*=6.6, 2H), 3.19 (t, *J*=6.6, 2H), 1.83 (pent, *J*=7.3, 2H), 1.62 (pent, *J*=6.9, 2H), 1.43-1.29 (m, 8H) ppm. ^13^C NMR (125 MHz, CDCl_3_): δ 138.7, 128.4 (2C), 127.6 (2C), 127.5, 72.9, 70.4, 33.5, 30.5, 29.7, 29.3, 28.5, 26.1, 7.3 ppm. EIMS *m/z* (%): 346.0789 (24, [M]^+^, calc’d 346.0794), 255.0272 (48), 131.0882 (45), 109.1081 (78), 108.0597 (51), 92.0711 (92), 91.0718 (100), 67.0573 (57); ESI HRMS [M + H]^+^ calc’d for C_15_H_24_IO: 347.0872, found: 347.0887.

Synthesis of ((tetradec-9-yn-1-yloxy)methyl)benzene (**5**)

A solution of *n*-BuLi (1.6M, 1.08 mL, 1.73 mmol) was added drop wise to a stirred solution of 1-hexyne (239 μL, 2.08 mmol, 1.2 eq) and HMPA (42.5 μL, 2.44 mmol) in dry THF (2 mL) cooled to -70 °C under Ar. The solution was warmed to -10 °C and then cooled again to ‑70 °C, where a solution of **4** (600 mg, 1.73 mmol, 1.0 eq) in THF (1 mL) was cannulated into the cooled solution. The solution was brought up to -30 °C and finally ambient temperature where it was left stirring for 3 days under an inert atmosphere. The reaction was quenched with water and extracted with hexane. The organic layer was washed with brine, water and dried over MgSO_4_. The organic layer was filtered through a silica plug and rinsed with excess hexane. The solvent was removed under reduced pressure to yield a yellow oil (375 mg, 1.25 mmol, 72% yield). IR (film) ν: 3033, 2929, 2858, 2106, 1458, 1365, 1104, 739, 702 cm^-1^. ^1^H NMR (500 MHz, CDCl_3_): δ 7.34 (d, *J*=8.5, 2H), 7.33 (m, 3H), 4.50 (s, 2H), 3.47 (t, *J*=6.6, 2H), 2.14 (m, 4H), 1.62 (m, 4H), 1.51-1.27 (m, 12H), 0.92 (t, *J*=7.1, 3H) ppm. ^13^C NMR (125 MHz, CDCl_3_): δ 138.7, 128.4, 127.6 (2C), 127.5 (2C), 80.2 (2C), 72.9, 70.5, 31.3, 29.8, 29.7, 29.4, 29.2, 29.1, 28.8, 26.2, 18.8, 18.5, 13.7 ppm. EIMS *m/z* (%): 300.2422 (15, [M]^+^, calc’d 300.2453), 107.0631 (64), 95.0834 (62), 92.0684 (68), 91.0652 (100), 81.0741 (70), 79.0579 (62), 67.0584 (71); ESI HRMS [M + H]^+^ calc’d for C_21_H_33_O: 301.2531, found: 301.2528.

Synthesis of *cis*-9-Tetradecen-1-ol (**6**)

Quinoline (128 μL, 1.08 mmol) and Lindlar’s catalyst (64 mg) was added to a stirred solution of **5** (92 mg, 0.31 mmol) in anhydrous methanol (6.4 mL) and allowed to react under an atmosphere (1 atm) of hydrogen for 8 hours at ambient temperature. The resulting suspension was filtered through Celite and thoroughly washed with diethyl ether. The filtrate was sequentially washed with a 1 M HCl (3 x 2 mL), a saturated CuSO_4_ solution (3 x 2 mL), brine (2 x 2 mL) and finally deionized water (2 mL). The organic layer was dried over MgSO_4_, filtered and the solvent removed under reduced pressure. Compound **4** was purified by semi-preparative gradient NP-HPLC (100 % hexane over 5 min then ramped to 30% EtOAc over 12 min. The gradient was then ramped to 100% EtOAc which was held for 5 min on silica). This purification was followed by isocratic (100% MeCN on C-18) analytical RP-HPLC affording a clear oil (32 mg, 0.15 mmol 48% yield, >99% purity by GC). IR (film) ν: 3337 (br), 2925, 2857, 1462, 1048, 726 cm^-1^. ^1^H NMR (500 MHz, CDCl_3_): δ 5.36 (m, 2H), 3.51 (t, *J*=6.6, 2H), 2.10-2.05 (m, 4H), 1.62 (m, 4H), 1.51-1.27 (m, 12H) 0.88 (t, *J=*7.0, 3H) ppm. ^13^C NMR (125 MHz, CDCl_3_): δ 130.6, 130.3, 60.4, 32.6, 32.6, 32.3, 29.7, 29.6, 26.2, 22.7, 22.5, 22.3 21.0, 13.7ppm. EIMS *m/z* (%): 96.3356 (54), 95.0849 (55), 82.0751 (77), 81.0694 (81), 69.0604 (66), 67.0541 (96), 55.0548 (100); ESI HRMS [M + H]^+^ calc’d for C_14_H_29_O: 213.2218, found: 213.2216.

**Calculation of Compound Ratios in Egg Extract**

A standard plot was produced using an Agilent 7980A GC interfaced to an Agilent 7000 series QqQ mass spectrometer (Agilent Technologies, Santa Clara, CA, USA) operating in electron ionization (EI) mode. One microliter injections of 0.005, 0.01, 0.025, 0.05, 0.1, 0.25, 0.5, 1.0 and 2.5 µL/mL of compounds **1**-**4** were vaporized on the preheated split-less inlet at 360 °C and introduced onto an SLB-5ms column (30 m × 0.25 mm i.d., 0.25 µm film thickness, Supelco 28471-U) using a 20 min temperature gradient (initial oven temperature of 150 °C, held for 4 min, heated to a final temperature of 230 °C at a rate of 4 °C/min, then held at final temperature for a further 3 min). Helium was used as a carrier gas at a constant flow rate of 1 mL/min. The peaks were integrated for each compound and a standard curve plotted (Figure 1). All concentrations were adjusted for purity. These plots were used to determine the natural ratio of compounds **1**-**4** present in the original egg extract. The artificial blend was ultimately created at a ratio of 7.8 : 2.3 : 2.3 : 1.0 for pentadecene, hexadecene, *cis*-9-tetradecen-1-ol and tridecene respectively.

**Figure 1:** A representative standard plot for determining the concentration of pentadecene in the egg extracts. The linear equation and R-squared value inset.

1. Wyckoff RWG (1922) The crystal structure of silver oxide (Ag_2_O). American Journal of Science 3: 184-188.
